# Generalized Coupled Cluster Theory for Ground and Excited State Intersections

**DOI:** 10.1021/acs.jpclett.4c03276

**Published:** 2025-01-07

**Authors:** Federico Rossi, Eirik F. Kjønstad, Sara Angelico, Henrik Koch

**Affiliations:** Department of Chemistry, Norwegian University of Science and Technology, NTNU, 7491 Trondheim, Norway

## Abstract

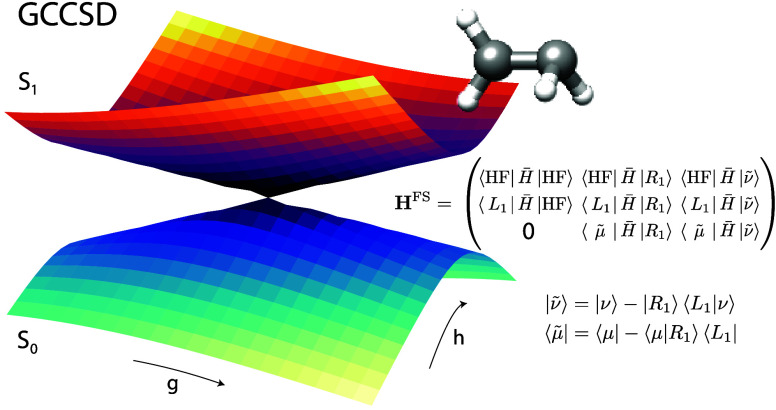

Coupled cluster theory in the standard formulation is unable to correctly describe conical intersections among states of the same symmetry. This limitation has restricted the practical application of an otherwise highly accurate electronic structure model, particularly in nonadiabatic dynamics. Recently, the intersection problem among the excited states was fully characterized and resolved. However, intersections with the ground state remain an open challenge, and addressing this problem is our objective here. We present a generalized coupled cluster framework that correctly accounts for the geometric phase effect and avoids bifurcations of the solutions to the ground state equations. Several applications are presented that demonstrate the correct description of ground state conical intersections. We also propose how the framework can be used for other electronic-structure methods.

Molecular systems with quasi-degeneracies or conical intersections between the ground and excited states present a significant challenge for single-reference coupled cluster methods. Although numerous multireference coupled cluster methods have been proposed over the past 40 years, comprehensive assessments indicate that no satisfactory solution has yet been found.^1−3^ As a result, coupled cluster methods have not been used to describe ground state conical intersections, even though such degeneracies are critically important to nonradiative relaxation processes found in a wide range of biological and chemical systems.^4^ In this paper, we do not aim to solve the general multireference case, which for instance is needed to describe the dissociation of molecules, limiting ourselves to the specific case of conical intersections between the ground and excited states.

The description of excited state intersections is also flawed in standard coupled cluster theory,^5−7^ except for the geometric phase effect.^8^ However, it is now known that the problems associated with excited state intersections (distortion of potential energy surfaces, complex energies, and incorrect topology) can be corrected by enforcing orthogonality relations between the electronic states. This is the main idea behind similarity constrained coupled cluster (SCC) theory,^9,10^ which provides a small correction to standard coupled cluster theory that restores a correct description of conical intersections. This method was recently applied successfully in nonadiabatic dynamics simulations on gas-phase thymine,^11,12^ opening up a range of applications to excited-state relaxation processes.

Recently Kjønstad and Koch^13^ demonstrated that the ground state coupled cluster wave function fails to account for the geometric phase encountered when traversing a path around a conical intersection. This leads to divergences in the coupled cluster wave function and results in a multivalued potential energy surface, where different surfaces can arise depending on the direction of the path taken around the conical intersection. As shown in ref (13)., these divergences are not only confined to small regions of the potential energy surface but extend throughout the entire configuration space encircling a ground state conical intersection.

Another complication arises when the Jacobian matrix becomes nearly singular, which occurs near the intersection. This situation defines a bifurcation point^14^ in the amplitude equations, leading to multiple possible solutions. These solutions have been studied in great detail previously.^15−17^ However, to the best of our knowledge, a wave function parametrization that eliminates these bifurcations has not been proposed. Each of the multiple solutions may be a reasonable approximation in some regions but completely unphysical in others, and there can be regions of internal coordinate space where the amplitude equations cannot be solved when the amplitudes are restricted to be real. We will show examples of these cases below.

The study of regions near ground state conical intersections in coupled cluster theory is highly challenging due to the bifurcation of solutions and the breakdowns caused by the phase effect.^13^ This is probably the reason why this area is largely unexplored in the community. The algorithm recently described by Angelico, Kjønstad, and Koch^18^ is therefore an indispensable tool when exploring the configuration space near intersections. This algorithm determines structures on an enveloped seam (also referred to as a tube) that is large enough to avoid the unphysical regions and sufficiently small to give reliable minimum energy conical intersection structures. These structures are denoted as ε-MECI, where ε corresponds to the extent of the tube that is wrapped around the seam.

Several approaches have been developed to circumvent the ground state intersection problem in electronic structure methods. These methods mainly involve starting from a different state of the system, for instance, a high-spin triplet state followed by a spin-flip (SF) in the equation-of-motion (EOM) treatment (SF-TD-DFT^19^ and EOM-SF-CCSD^20^) or starting from a double ionized state and adding electrons back in via the EOM framework (hh-TDA^21,22^ and DEA-EOM-CCSD^23,24^). Other methods, notably in density functional theory (DFT), have been developed that either change the kernel to account for double excited states^25^ or by adding an additional double excited configuration to the Hermitian Tamm-Dancoff eigenvalue problem (TDDFT-1D^26^). Ensemble DFT (eDFT) methods have also been applied to strongly correlated systems and extended to treat excited states (SI-SA-REKS^27^). More recently, Schmerwitz et al.^28^ developed an algorithm to determine saddle points in DFT methods that can be identified with electronic excited states, giving access to ground state intersections. This method is related to the norm-extended optimization scheme for multiconfigurational wave functions^29^ that also determine saddle point states that are nonorthogonal.

In this work, we derive a generalized coupled cluster theory (GCC) that avoids all the unphysical behaviors mentioned above. The framework corrects the ground state wave function parametrization and does not consider a different state of the system as a starting point. The complications related to matrix defects in the non-Hermitian eigenvalue problem can be handled using the techniques developed in similarity-constrained coupled cluster theory and will not be discussed here. The application of GCC with singles and doubles excitations (GCCSD) will be illustrated for several molecular systems where the coupled cluster singles and doubles (CCSD) model fails to give a correct description. We will also formulate the GCC2 model, which is a generalization of the well-established CC2 model.^30^ Finally, we will propose a procedure to eliminate bifurcations in Hartree–Fock and density functional theory, which in their present formulation are unable to describe ground state conical intersections.^31,32^

## Generalized Coupled Cluster Theory

In standard coupled cluster theory, the electronic wave function is given by
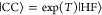
1where the cluster operator *T* is expressed in terms of excitation operators τ_μ_ and cluster amplitudes *t*_μ_, such that
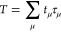
2The excitation operators, labeled by the index μ, are defined with respect to a closed-shell Hartree–Fock reference |HF⟩, and thus the operators commute among one another. The energy and the amplitudes are determined by projecting the electronic Schrödinger equation on {⟨HF|, ⟨μ|} and we obtain the well-known equations
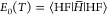
3
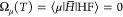
4where eq 4 is the amplitude equations and *E*_0_ is the coupled cluster energy.^33^ The similarity-transformed Hamiltonian is given by  = exp(−*T*)*H* exp(*T*), where *H* is the electronic Born–Oppenheimer Hamiltonian.

The amplitude equations can be expanded in a Taylor series in the following way
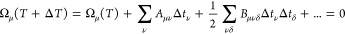
5where the Jacobian and its derivative are given by
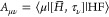
6
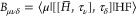
7If we assume the Jacobian is diagonalizable (**S**^–1^**AS** = **D**) we may write eq 5 in the eigenbasis
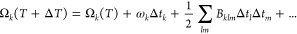
8where Ω_*k*_ = (**S**^–1^Ω)_*k*_, *Δt*_*k*_ = (**S**^–1^*Δt*)_*k*_, τ_*k*_ = (τ^*T*^**S**)_*k*_, and ω_*k*_ is an eigenvalue of **A**. It is clear that if ω_*k*_ is close to zero, then the higher-order terms in the expansion dominate, giving rise to a bifurcation into more than one solution. Depending on the properties of these higher-order terms, we may obtain different situations with two or more real solutions or even none. Before we discuss our solution to the bifurcation problem, let us consider the equation of motion eigenvalue problem^34,35^ for 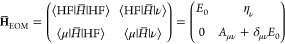
9where we assume the amplitudes equations have been solved (Ω_μ_ = 0). From eq 9 we observe the excitation energies are equal to the eigenvalues of the Jacobian **A**. We introduce the following notation
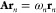
10
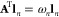
11where the left and right eigenvectors are biorthonormal, that is, **l**_*m*_^*T*^**r**_*n*_ = δ_*mn*_. Furthermore, the left and right states are written as
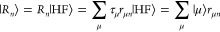
12
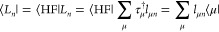
13and we define the biorthogonal projection operators as
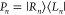
14When **A** is diagonalizable this set of projectors is complete, that is ∑_*n*_*P*_*n*_ = 1. We can now expand the amplitudes in this basis
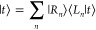
15where |*t*⟩ = ∑_ν_ |ν⟩*t*_ν_. From the numerical investigation of the CCSD model presented in the Application section, we observe that, for certain solutions to the ground state equations in eq 4, components of the cluster amplitudes, specifically ⟨*L*_*n*_|*t*⟩, can become very large and may even exhibit diverging behavior. We therefore propose to remove these components from the cluster amplitudes and solve the amplitude equations for a restricted set of amplitudes. When projecting out the lowest eigenvector, the effective Jacobian that enters the amplitude equations becomes positive definite and we obtain a convex problem without a bifurcation. This is the basic idea behind the generalized coupled cluster theory we will outline.

We will initially formulate GCC when projecting only one state. The extension to several states is straightforward and is shown in the Supporting Information. We denote this state as ⟨*L*_1_| and |*R*_1_⟩, with the associated Jacobian eigenvalue ω_1_, and we introduce the modified projection manifold
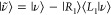
16
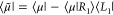
17We have that ⟨*L̃*_1_| = ∑_μ_*l*_μ1_⟨μ̃| = 0 and similarly |*R̃*_1_⟩ = 0, as ⟨*L*_1_|*R*_1_⟩ = 1. Working with this set is more convenient, as the block structure of the matrices becomes more transparent. Alternatively, we could have used {|HF⟩, |μ⟩}, for both the left and right basis. We now require that the cluster amplitudes do not contain the eigenvector components
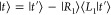
18and we determine the remaining amplitudes such that
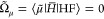
19
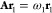
20
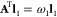
21These are coupled sets of equations that we solve using standard techniques. Details about the convergence are presented in the Application section. It is clear that as we have removed one component from the cluster operator, we cannot in general obtain the full configuration interaction solution for the ground state coupled cluster wave function. However, we may include the projected components in the diagonalization of the similarity-transformed Hamiltonian and thereby obtain the ground and excited states, also in the exact limit. Employing the following left basis {⟨HF|, ⟨*L*_1_|, ⟨μ̃|} and right basis {|HF⟩, |*R*_1_⟩, |ν̃⟩}, we obtain the full space eigenvalue equations
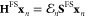
22
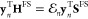
23where the matrices are defined as
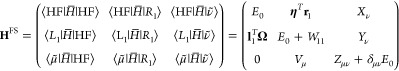
24
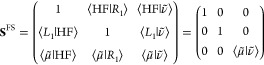
25and the terms in eq 24 are implicitly defined. Further information can be found in the Supporting Information. In the metric matrix **S**^FS^, the overlap between the left and right projected bases is given by ⟨μ̃|ν̃⟩ = δ_*μν*_ – ⟨μ|*R*_1_⟩⟨*L*_1_|ν⟩. Although we can only obtain the full configuration interaction limit in the full space case, it is instructive to consider the 2 × 2 reduced space matrix
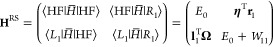
26In Applications, we numerically demonstrate that the reduced matrix **H**^RS^ is an excellent approximation to the corresponding full space eigenvalues. This suggests we can bypass solving the full space eigenvalue equation, reducing the computational cost of the framework.

We note that solving the eigenvalue problem for both the reduced matrix and the full matrix can sometimes result in a complex pair of eigenvalues and eigenvectors, which is what is observed in a small region close to the conical intersection (see Figure 2 and Figure S4). This situation is discussed in ref (7). for the case of conical intersections among excited states, but the same conclusions can be applied to the problem in eq 22. This final step presents the same characteristics as EOM-CC, where the partial ground state solution obtained in the reduced parameter space acts as the ground state, and the Jacobian matrix is replaced by the full space matrix.

Before investigating the scaling properties of GCC, a few observations about the geometric phase are in order. In the presence of a conical intersection between the ground and first excited states, the wave functions should exhibit a geometric phase when traversing around the intersection. However, when we exclude the excited state from the cluster amplitudes, we expect that the amplitudes will no longer display a geometric phase. Similarly, the eigenvectors of the Jacobian should also not exhibit any phase. On the other hand, the states obtained by diagonalizing the non-Hermitian eigenvalue problem in eq 22 will exhibit a correct geometric phase effect, as explained by Williams et al.^8^ This is confirmed numerically below.

## Scaling with System Size

Size extensivity of total energies and size intensivity of excitation energies are two essential properties of coupled cluster theory that ensure scalability to large systems without losing accuracy.^33,36^ Therefore, we will show that these properties are maintained in GCC. We consider a system composed of two noninteracting subsystems A and B. The total Hamiltonian is the sum of two separate terms *H* = *H*_*A*_ + *H*_*B*_. When the cluster amplitudes are extensive, *T* = *T*_*A*_ + *T*_*B*_, we have that  =  +  where  = exp(−*T*_*X*_)*H*_*X*_ exp(*T*_*X*_). The Hartree–Fock reference is given by the direct product state |HF⟩ = |HF_A_⟩ ⊗ |HF_B_⟩ and the excitation manifold is ordered as {⟨μ_*A*_|, ⟨μ_*B*_|, ⟨μ_*AB*_|}.

It is well-known that when the cluster amplitudes are extensive then the right eigenvectors of the Jacobian in eq 21 are intensive.^36^ Thus, we consider a right eigenvector located in system A and denote the excitation operator *R*_1_^*A*^. The corresponding left operator *L*_1_^*A*^ does not have components in B but can have nonzero elements in the AB part of the operator. We first note that the amplitude equations in eq 20 take the form
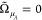
27
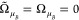
28
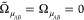
29where we have used that ⟨μ̃_*B*_| = ⟨μ_*B*_| and ⟨μ̃_*AB*_| = ⟨μ_*AB*_|. We conclude there is a size-extensive solution as the AB part is always zero whenever the cluster amplitudes are extensive. We can now analyze the structure of the full space Hamiltonian matrix
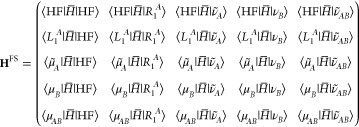
30where we have used that |ν̃_*B*_⟩ = |ν_*B*_⟩. Using eqs 27-29 we may show that
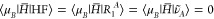
31
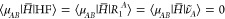
32and this leads to the following block structure of the matrix
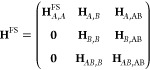
33The full space matrix in the A system is given by
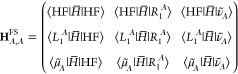
34and the other matrix elements in eq 33 are implicitly defined. Further analysis of this matrix reveals that
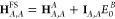
35where **H**_*A*,*A*_^*A*^ is obtained from eq 34 replacing  with  Thus, we have shown that the total energies in A are size-extensive and the excitation energies are size-intensive as the diagonal elements are shifted with ground state energy of the B system.

We now consider size-extensivity in the B system and introduce another system C that is noninteracting with both A and B. We consider the matrix
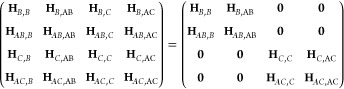
36and we observe that all the new coupling blocks are zero, giving size-extensivity and size-intensity in the B and C systems. We should point out, that the *E*_0_^*A*^ is not equal to the ground state energy obtained from eq 34, since Ω_μ_*A*__ ≠ 0. This also implies that the coupling blocks, for instance **H**_*AB*,*B*_, modify the excitation energies in B and C. In the case where we have sufficiently high excitations to obtain full configuration interaction (FCI) in both A and B or in A and C, the modifications from the coupling blocks provide the FCI excitation energies in B or C. These errors are tiny and do not scale with the overall system size because of the block structures outlined above. Numerical examples will be presented in the Application section. We note that if Jacobian eigenvectors from different subsystems are projected simultaneously, the resulting energies will not be extensive and the errors will scale with the number of subsystems projected.

## Applications

**Figure 1:**
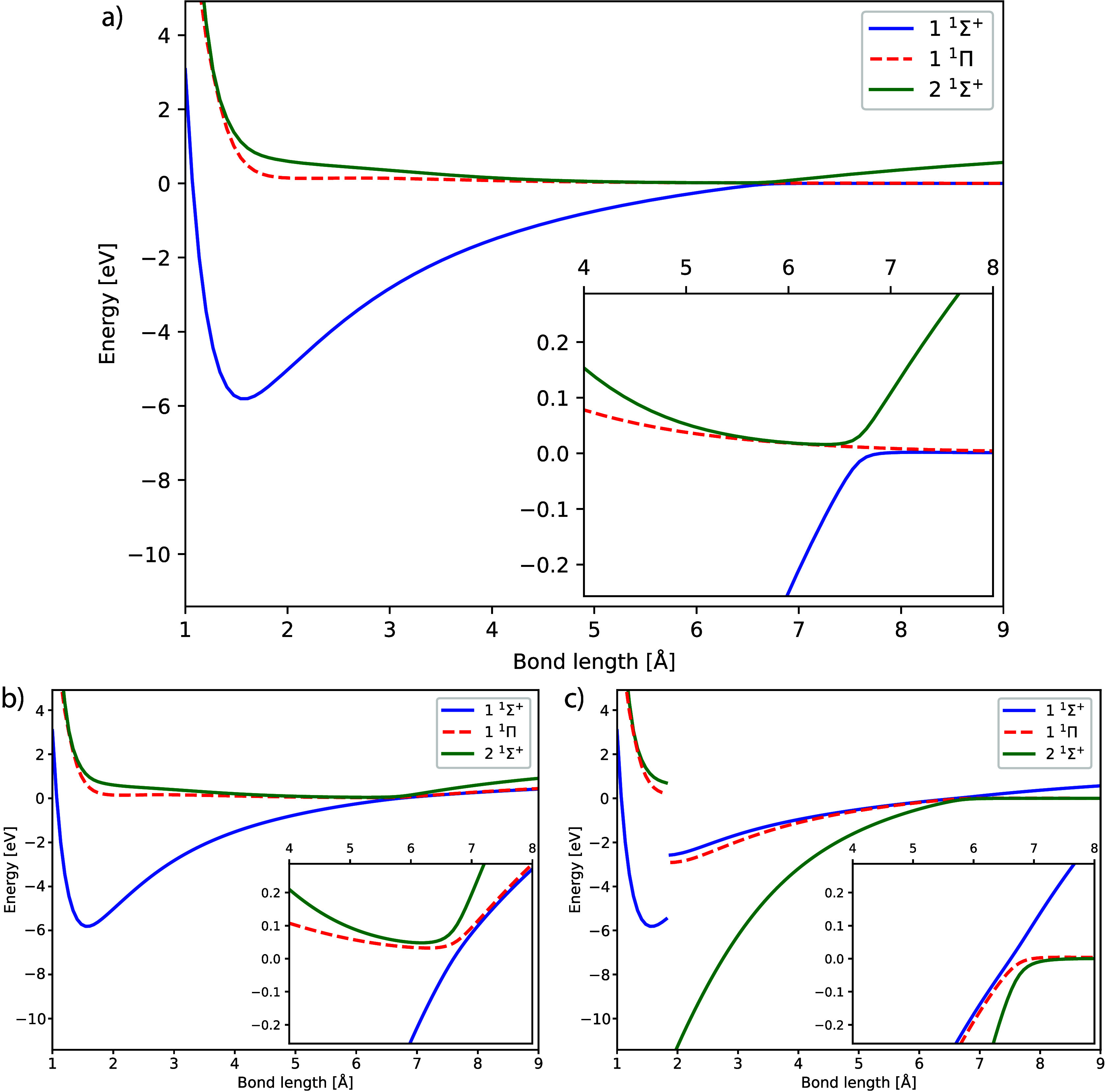
(a) GCCSD energy levels of LiF at different interatomic distances while projecting 3 states. The CCSD results are reported in (b) starting from 1.0 Å and increasing the interatomic distance and in (c) starting from 9.0 Å and decreasing the interatomic distance. The inset shows a close-up view of the avoided crossing.

The GCCSD framework, for an arbitrary number of projected states, was implemented in a local development version of the *e*^*T*^ program^37^ and used in all the calculations reported here. Initially, we will consider the lithium fluoride molecule, a standard benchmark system for multireference coupled cluster methods.^38^ At a bond distance of about 6.75 Å the molecule has an avoided crossing^39^ between the 2^1^Σ^+^ state and the ground state 1^1^Σ^+^. We employ an aug-cc-pVDZ basis and find two solutions to the CCSD ground state equations. The CCSD solution (Figure 1b), obtained when starting at distances close to the equilibrium bond length (1.5 Å), shows an unexpected increasing diverging trend in the energies of all 3 states when extended past the avoided crossing and restarting the algorithm from the previous point. Another CCSD solution is found (Figure 1c) when starting at a long bond distance (9.0 Å). We observe a decreasing diverging trend in the energies when extended to shorter distances. The solution eventually changes discontinuously to the other CCSD solution at about 2.0 Å. On the other hand, the GCCSD curves shown in Figure 1a are continuous for the whole range of bond distances. Thus, GCCSD bridges the CCSD solution for distances shorter than the avoided crossing with the other CCSD solution beyond the avoided crossing point. In doing so, GCCSD corrects the divergent behavior seen in the two individual CCSD solutions.

**Figure 2:**
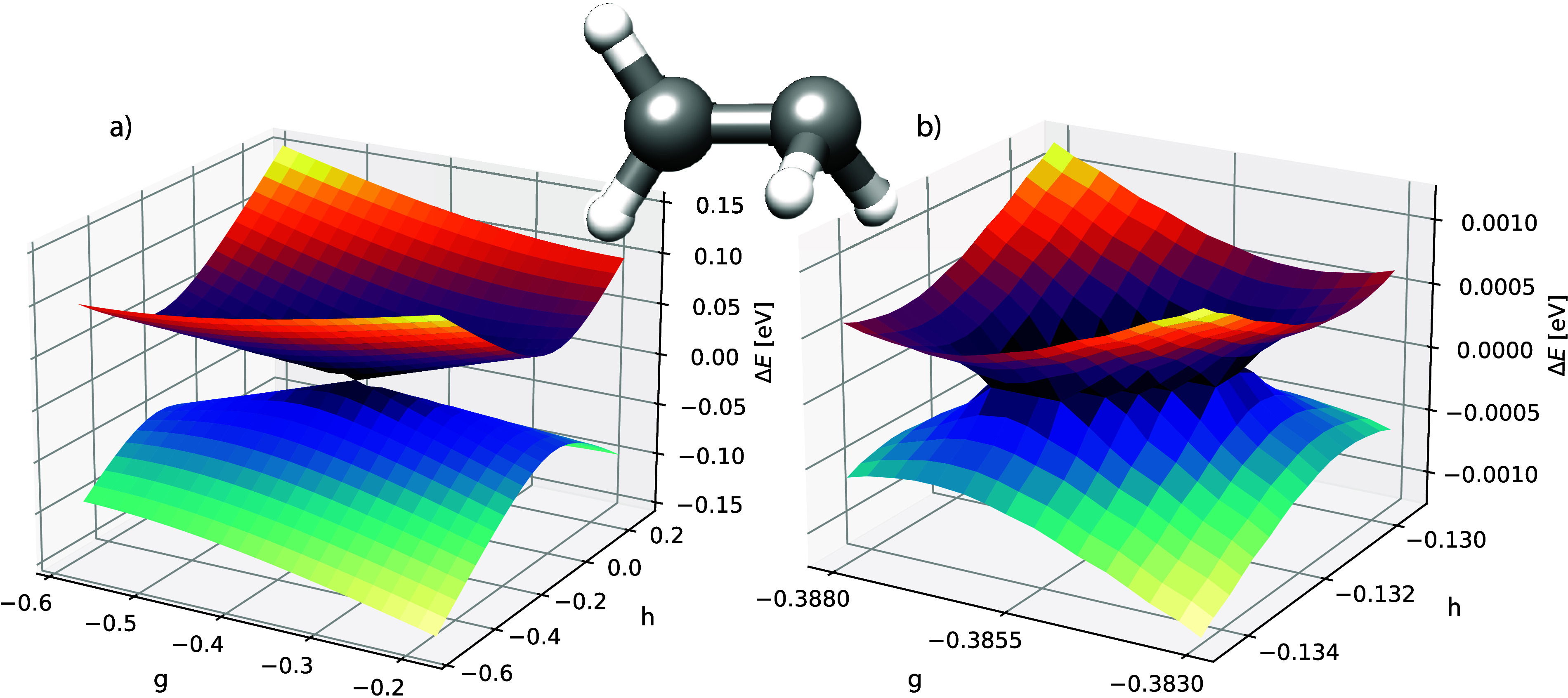
(a) The GCCSD potential energy surfaces of S_0_ and S_1_ in ethylene. (b) A detailed view of the region close to the conical intersection. The basis is aug-cc-pVDZ, and for each point the energies are plotted relative to the average energy 1/2(*E*_0_ + *E*_1_). A plot of the same region, showing the absolute energies of the two states in Hartree, can be found in the Supporting Information. The ε-MECI structure is shown in the middle, which corresponds to the geometry at the point (g, h) = (0.0, 0.0).

We now consider a ground state conical intersection in ethylene. We will start from the CCSD ε-MECI using an aug-cc-pVDZ basis to explore the potential energy surfaces close to a conical intersection. The ε-MECI structure is reported by Angelico et al.^18^ and is shown in Figure 2. We further employ the **g** and **h** vectors calculated at the ε-MECI geometry using the CCSD algorithm described in ref (40). Further computational details are reported in the Supporting Information.

**Figure 3:**
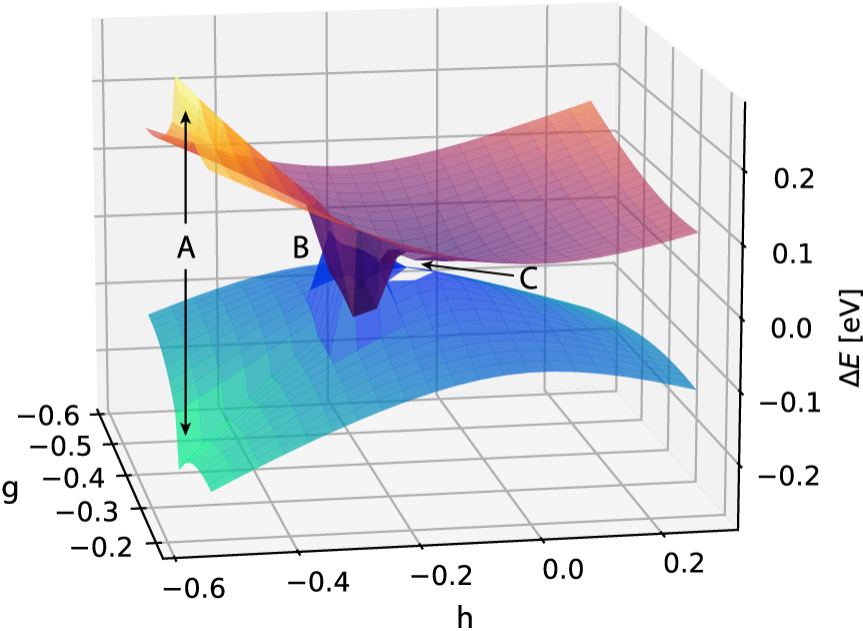
CCSD potential energy surfaces of S_0_ and S_1_ in ethylene. The basis is aug-cc-pVDZ and for each point the energies are plotted relative to the average energy 1/2(*E*_0_ + *E*_1_). There are three notable regions: in **A**, a mismatch in energies appears due to the phase effect; in **B**, a new set of flipped solutions is obtained, characterized by negative excitation energies; and in **C**, the region where we were not able to converge the ground state equations.

In Figure 2a we show a GCCSD conical intersection between S_0_ and S_1_. When we zoom in on the intersection region, shown in Figure 2b, we observe a small defective area where the full space matrix has complex energies. Aside from a minor defect, the intersection exhibits the correct conical shape. This is the expected behavior and is explained in earlier works.^7^ However, when compared to a typical CCSD calculation in the branching plane, the difference is striking, as shown in Figure 3, which clearly illustrates the breakdown of the CCSD model. Here we have mismatches in energies due to problems describing the geometric phase,^13^ the bifurcation of the solution resulting in regions with negative excitation energies, and a region where we were not able to converge the ground state equations.

**Figure 4:**
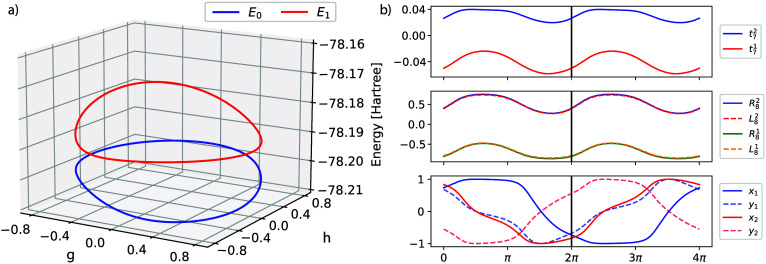
GCCSD potential energy curves of S_0_ and S_1_ in ethylene (a), when traversing on a circle around the conical intersection, using aug-cc-pVDZ. In (b), some selected GCCSD parameter values are reported for a 4π rotation around the intersection. Starting from the top, the two largest components of the cluster amplitudes, the two largest components of the left and right eigenvectors, and finally the 2 eigenvectors of the reduced matrix, where (*x*_1_, *y*_1_) refer to S_0_ and (*x*_2_, *y*_2_) to S_1_.

We now investigate the geometric phase effect in GCCSD. In Figure 4a we have mapped out the potential energy curves of S_0_ and S_1_ when traversing a circle around the conical intersection. The Supporting Information provides more details on how these plots are generated. We observe that both curves are continuous without any artifacts. In Figure 4b we examine the behavior of the parameters during a 4π rotation. We see that the cluster amplitudes and the eigenvectors of the Jacobian are unchanged after a 2π rotation. The eigenvectors of the reduced space matrix display the geometric phase effect and change sign at 2π and return to the original value after 4π. Thus, we conclude that GCC correctly accounts for the geometric phase effect.

**Figure 5:**
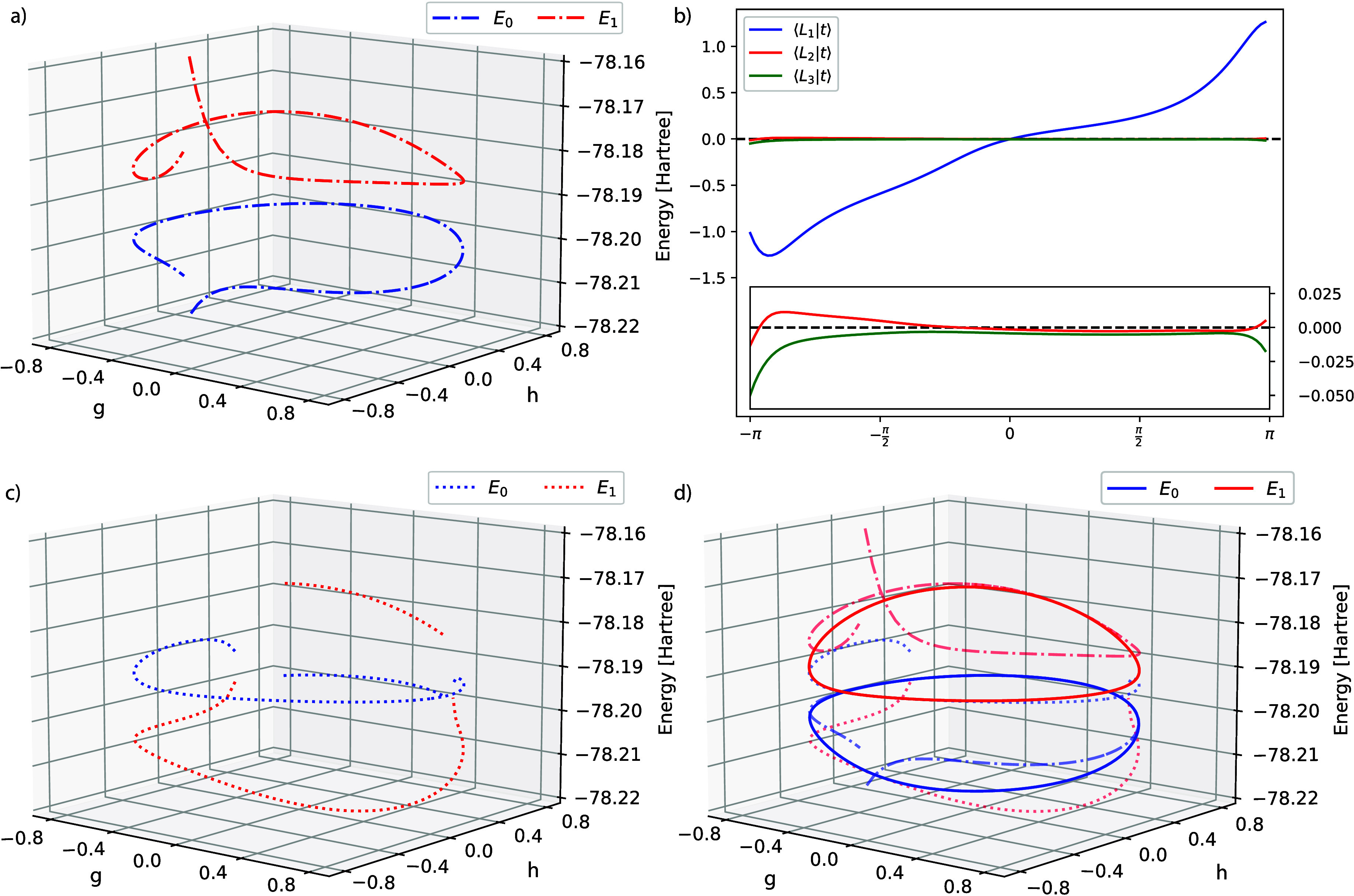
CCSD potential energy curves of S_0_ and S_1_ in ethylene, when traversing on a circle around the conical intersection, using aug-cc-pVDZ. The two sets of curves in (a) and (c) have been obtained starting from opposite points, (*g*, *h*) = (0, ± 0.8) respectively, and restarting from the previous point when mapping out a half circle in both directions. In (b) is shown the projection of the amplitudes on the 3 lowest left eigenvectors for the solution in (a) when rotating ± π. In (d) the same curves are plotted together with the GCCSD energies (solid line) shown in Figure 4a.

In Figure 5 we present the corresponding CCSD curves. In Figures 5a and 5c we have mapped out the two different solutions that are obtained due to the bifurcation in the ground state equations. The solution shown in Figure 5a encounters problems as we approach the point where the weight of the Hartree–Fock reference becomes zero–this situation is discussed in detail in ref (13). The other solution is shown in Figure 5c where the ground state solution is close to S_1_ and the excitation energy is negative. As we traverse the circle, the solution encounters a region where the equations do not converge, followed by a change to the solution shown in Figure 5a. In Figure 5b we show the different components of the CCSD amplitudes and observe the diverging behavior of the component along *L*_1_. In Figure 5d we have shown GCCSD together with the two CCSD solutions. We observe that CCSD is reasonably close to GCCSD in some regions of the circle but is far away in others.

To demonstrate the size-intensivity of GCCSD excitation energies, we consider a system of three ethylene molecules in similar but distinct geometries: A, B, and C. The molecules are spaced 1000 Å apart, ensuring they do not interact with each other. In the first column of Table 1, the first two CCSD excitation energies are reported for the 3 isolated molecules. The second column reports the GCCSD excitation energies on system A alone where we project the first excited state. When system B is included (see the third column), the projected state is localized on A, and system A’s excitation energies are unaffected. Excitations in B are only slightly modified, due to the coupling block **H**_*AB*,*B*_ in eq 33. When an additional noninteracting ethylene molecule C is added, excitation energies in both A and B are unaffected, as expected from the block structure in eq 36. In the Supporting Information, we report additional cases that illustrate the scaling properties.

**Table 1:** Two Lowest Excitation Energies of Ethylene in 3 Similar but Different Geometries (A, B and C)^a^

System	CCSD X	GCCSD A	GCCSD AB	GCCSD ABC
*ΔE*_1_^*A*^	0.0168 318 946	0.0168 301 978	0.0168 301 976	0.0168 301 975
*ΔE*_1_^*B*^	0.0172 860 929	-	0.0172 863 677	0.0172 863 676
*ΔE*_1_^*C*^	0.0177 393 431	-	-	0.0177 396 178
*ΔE*_2_^*A*^	0.1511 668 829	0.1511 672 134	0.1511 672 136	0.1511 672 135
*ΔE*_2_^*B*^	0.1512 823 538	-	0.1512 826 298	0.1512 826 296
*ΔE*_2_^*C*^	0.1514 009 837	-	-	0.1514 012 593

a For all GCCSD calculations, only one state is projected, which is always localized on A. In the two last columns, the system is composed of multiple ethylene molecules in different geometries and shifted by 1000 Å so that they do not interact with each other. All calculations are performed with aug-cc-pVDZ, with convergence threshold of 1 · 10^–10^.

In concluding our investigation of ethylene, we discuss the convergence properties of the framework. We use the ethylene A geometry from the extensivity study above, given in the Supporting Information. In Table 2 we report the energies for the ground state and the two first excited states. The reduced space numbers are almost identical to the full space and in turn, these are similar to the CCSD numbers. Even though the number of iterations indicates a factor of 2.5 in computational cost between GCCSD and CCSD, the wall times show only a factor of 1.7. This is due to relatively fewer linear transformations in GCCSD compared to CCSD per iteration.

**Table 2:** Energies and Information about Convergence for One Ethylene Molecule^a^

System	Energy		Iterations	Time
CCSD	*E*_0_	–78.1978 872 879	33	57.0 s
	ω_1_	0.0168 318 946	33 + 33	
	ω_2_	0.1511 668 829		
GCCSD	*E*_0_^FS^	–78.1978 872 787	25	96.3 s
	*E*_0_^RS^	–78.1978 872 810		
	ω_1_^FS^	0.0168 301 977	101 + 104	
	ω_1_^RS^	0.0168 302 023		
	ω_2_^FS^	0.1511 672 134	27	

a Both CCSD and GCCSD calculations determine three excited states with a threshold of 1.0 × 10^–10^. For GCCSD, the first excited state is projected. The numbers of iterations reported are, from top to bottom: the number of DIIS iterations to solve the CCSD ground state equations, and the total number of Davidson’s solver iterations to obtain the right and left eigenvectors of the CCSD Jacobian. For GCCSD the number of DIIS iterations to solve the cluster amplitude equations. The number of iterations to find the right and left eigenvectors of the GCCSD Jacobian. Finally, the number of Davidson’s iterations to determine the right eigenvectors of the full matrix. The timings are wall times in seconds for the entire calculation on an Intel Xeon Gold 6342 using 24 cores.

**Figure 6:**
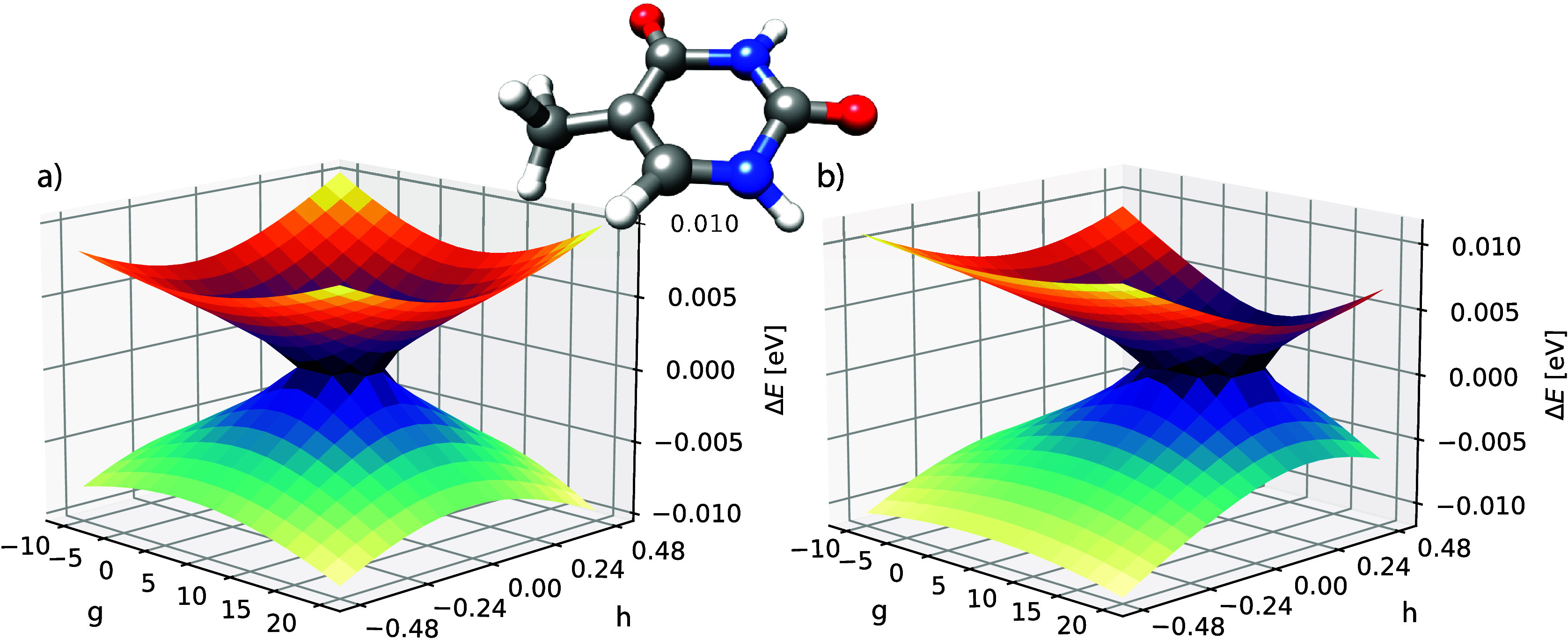
CCSD (a) and GCCSD (b) potential energy surfaces of S_1_ and S_2_ in thymine with cc-pVDZ basis. All energies are plotted in eV, relative to the average 1/2(*E*_1_ + *E*_2_) for each point. The conical intersection structure is shown in the middle, which corresponds to the geometry at the point (g, h) = (0.0, 0.0).

To demonstrate the GCCSD method when projecting out several states we consider the S_1_/S_2_ conical intersection in thymine. We project out the eigenvectors for the two lowest eigenvalues of the Jacobian. This means the reduced space matrix is a 3 × 3 matrix. We use an initial structure together with **g** and **h** vectors that were determined in ref (12). These are reported in the Supporting Information. In Figure 6 we show the S_1_ and S_2_ potential energy surfaces for CCSD and GCCSD. The surfaces look very similar and they both have a defect that can be removed using a similarity-constrained transformation, as shown in Figure S3 for CCSD. The center of the defect is slightly shifted when comparing the two.

**Figure 7:**
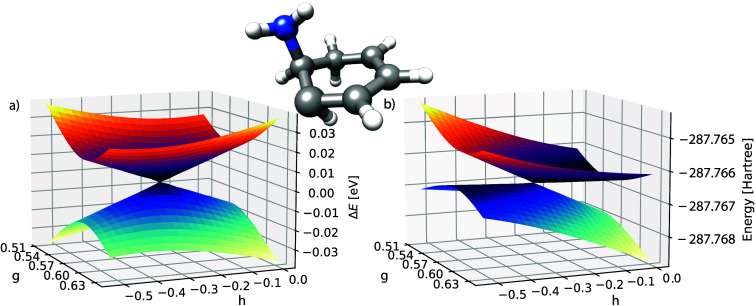
GCCSD potential energy surfaces of S_0_ and S_1_ in 2,4-cyclohexadien-1-ylamine with cc-pVDZ. In (a) the energies for each point are plotted in eV relative to the average energy 1/2(*E*_0_ + *E*_1_), whereas in (b) the total energies are shown in Hartree. The initial structure is shown in the middle, which corresponds to the geometry at the point (g, h) = (0.0, 0.0).

As a final example, we show a S_0_/S_1_ intersection in 2,4-cyclohexadien-1-ylamine. The geometry was obtained from the database in ref (41). and the structure is shown in Figure 7. The **g** and **h** vectors are calculated using the CCSD algorithm described in ref (40). In the same figure, we show the two potential energy surfaces in two different representations. The conical shape of the intersection is visible in both plots. Again, when zooming in on the intersection region, a small defective area is observed (see Figure S2 in the Supporting Information).

## Further Perspectives

The framework presented above can be extended to other electronic structure methods with similar problems accounting for the geometric phase associated with conical intersections with the ground state. For example, time-dependent Hartree–Fock (TDHF) and time-dependent density functional theory (TDDFT) cannot describe the intersection because the ground and excited states are decoupled.^31,42^ Other methods such as the algebraic diagrammatic construction hierarchy (ADC)^43,44^ also do not include coupling to the ground state. The ADC framework relies on Møller–Plesset perturbation theory for the ground state wave function and as shown in ref (13). the perturbation series converge to an excited state close to the conical intersection. Recently, Taylor et al.^45^ discussed the failure of the ADC(2) model at ground state intersections.

**Figure 8:**
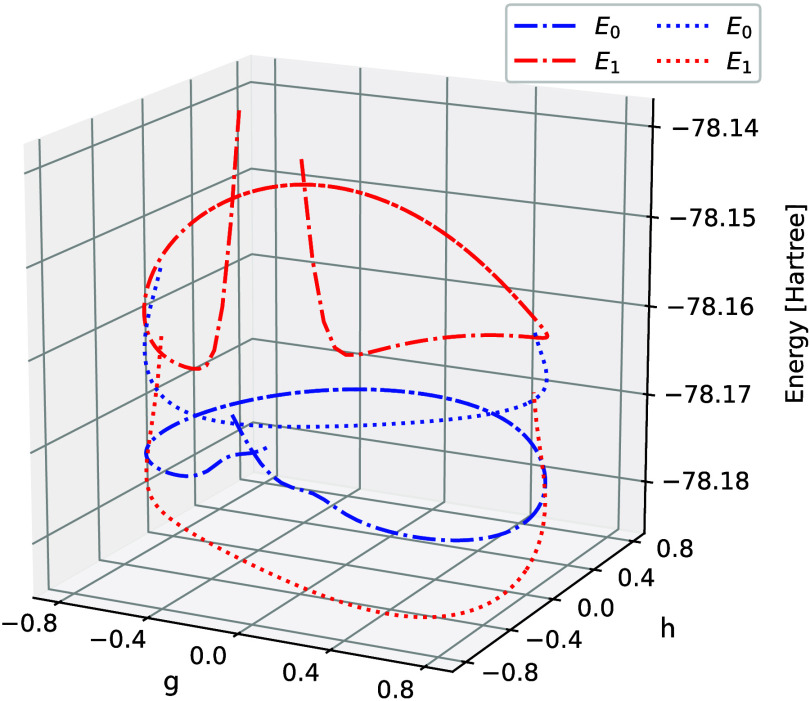
CC2 ground state and first excited state energies when traversing a circle around the conical intersection in ethylene, using aug-cc-pVDZ. The two sets of curves have been obtained starting from opposite points, (*g*, *h*) = 0, ±0.8 for the dash-dot and dotted lines respectively, and restarting half circle in both directions. The red dash-dot line is cut to limit the *z*-dimension, but a crossing between the two ends is present, similar to what can be seen for the blue dash-dot line.

The entire coupled cluster hierarchy is generally affected by the geometric phase issues highlighted in ref (13). Here we explicitly mention the CC2 method,^30^ which is frequently used for calculating excitation energies of large molecules, due to the favorable balance between computational cost and accuracy compared to CCSD. The CC2 model is viewed as the best alternative to second-order Møller–Plesset theory as both ground and excited states are available with the same computational cost and would therefore be an excellent candidate for nonadiabatic dynamics. However, the CC2 method also fails to describe ground state conical intersections, as shown in Figure 8 for the ethylene molecule. Using the GCC framework above, we may extend CC2 to GCC2 by considering the CC2 Hamiltonian matrix
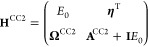
37The explicit expressions for **Ω**^CC2^ and **A**^CC2^ can be found in ref (30). We will report on the implementation of GCC2 elsewhere together with a detailed benchmarking of the method.

We now outline how the conical intersection problem could be solved for closed-shell Hartree–Fock. Due to the closed shell form of the Hartree–Fock wave function, a rotation of the orbitals cannot change the overall sign of the wave function. This implies that Hartree–Fock theory cannot detect an intersection with the excited state. However, as discussed in detail by Helgaker *et. al*,^33^ the eigenvalues of the electronic Hessian may become small and this can lead to bifurcations in the Hartree–Fock solution. Using the ideas from GCC we may remove these bifurcations. For this purpose, we parametrize the wave function in terms of orbital rotations
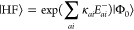
38where *E*_*ai*_^–^ = *E*_*ai*_ – *E*_*ia*_, *a*, *b* and *i*, *j* labels virtual and occupied orbitals, respectively, and |Φ_0_⟩ is the initial wave function. The gradient and Hessian are given by
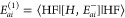
39
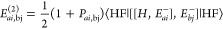
40where *P*_*ai*,*bj*_ permutes *ai* and *bj* indices. We may now remove the Hessian eigenvector **r** from the **κ** vector, which is associated with a small eigenvalue that gives rise to bifurcations. This leads to a coupled set of equations that must be solved in the same way as for GCC. The removed component may be included through diagonalization of the matrix
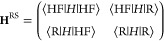
41where |R⟩ = ∑_*ai*_ |*ai*⟩*r*_*ai*_. The obtained solutions will be able to account for the geometric phase and describe a conical intersection between the two states. We should note that the energies will depend on the initial determinant |Φ_0_⟩, as we remove components from the orbital rotation operator. Furthermore, the Hartree–Fock wave function in eq 38 will not be exact for one electron. Therefore it would be more appropriate to include all single excitations in eq 41 and consider a full space matrix as in GCC.

In passing, we note that as the eigenvectors of the Hessian in eq 40 are size-intensive then the resulting wave functions will be extensive. They can therefore serve as a reference for a coupled cluster model that extends the GCC framework presented here. Needless to say, the framework can also be used for Tamm-Dancoff TDDFT.^31,32^

## Conclusions

In this paper, we have presented a coupled cluster framework capable of describing intersections with the ground state while accounting for the geometric phase effect and eliminating bifurcations in the ground state equations. This development paves the way for studying nonadiabatic dynamics among excited states with the possibility of also describing relaxation to the ground state. Indeed, we have already established techniques that can remove defects in the non-Hermitian eigenvalue problem. This similarity-constrained coupled cluster theory is directly transferable to GCC and analytical molecular gradients and derivative couplings can be calculated with minor modifications to the existing developments for CCSD and SCCSD.

Another aspect of the reported development concerns coupled cluster theory itself. In ref (13), we have recognized the need to move away from assuming that the coupled cluster ground state wave function is exact. Even the full coupled cluster wave function is not well-defined in an (*N* – 1) dimensional configuration space due to intermediate normalization. Therefore, instead of imposing exactness, we should focus on making sure that the coupled cluster wave function is well-behaved. The exact limit may instead be achieved by subsequent diagonalization of the similarity-transformed Hamiltonian matrix, which at the same time allows for a correct description of conical intersections with the ground state. As we have discussed, the approach also extends to other electronic structure theories, such as Hartree–Fock and density functional theory, opening up an interesting perspective for future developments in electronic structure theory.

## Supporting Information Available

Details on the implementation, algorithm, 2D and circular scans and the expressions of the reduced and full space Hamiltonian matrices for both one and multiple projected states; the ground and excited state energies of two noninteracting water molecules, thymine and thymine with a single helium atom when varying the number of projected states, and geometries, **g** and **h** vectors for all the systems considered (PDF)
