# Supplementary material for: Generalized Coupled Cluster Theory for Ground and Excited State Intersections
Source: J Phys Chem Lett. 2025 Jan 7;16(2):568–78. doi: 10.1021/acs.jpclett.4c03276 (PMC11744793; doi:10.1021/acs.jpclett.4c03276)
Supplement: Supplementary file 1 — jz4c03276_si_001.pdf [file jz4c03276_si_001.pdf]

Supporting Information for "Generalized coupled cluster theory for ground and excited state intersections"

Federico Rossi, Eirik F. Kjønstad, Sara Angelico, and Henrik Koch*

Department of Chemistry, Norwegian University of Science and Technology, NTNU, 7491 Trondheim, Norway

E-mail: henrik.koch@ntnu.no

Contents

Implementation **3**

Hamiltonian matrix in the new basis **3**

Single projected state . . . 3

Multiple projected states . . . 4

Algorithm and computational details **5**

Results on convergence and size extensivity **7**

Non-interacting identical water molecules . . . 7

Effect of the number of states in the projector . . . 8

Ethylene **10**

Ethylene 2D scan . . . 10

Ethylene circle . . . 11

Ethylene convergence . . . 12

Thymine 2D scan **16**

2,4-Cyclohexadien-1-ylamine 2D scan **18**

Implementation

A first version of the method was implemented in Julia, starting with a Hartree-Fock calculation using PySCF[1] to provide the integrals in the MO basis. The equations to construct omega and the product Jacobian times a vector was auto-generated using the Julia package `SpinAdaptedSecondQuantization.jl`[2] to provide the equations, which are then automatically converted into code based on np.einsums. A solver for the omega equations is implemented with DIIS acceleration to provide CCSD solutions, using `Arpack.jl` to iteratively solve the eigenvalue problem for the energies and vectors of the excited states. This code is used as a starting point to develop the GCCSD method, introducing the necessary projections, matrix construction and eigenvalue problem solvers. The results are tested on HeH_2 with STO-3G, to retrieve the same eigenvalues of an FCI reference calculation from eT.[3] For faster performance, a new version of the method is implemented in a local branch of eT, which is used for all the calculations reported in this work.

Hamiltonian matrix in the new basis

Single projected state

Reduced space Hamiltonian matrix

$$\mathbf{H}^{\mathrm{RS}} = \begin{pmatrix} \langle \mathrm{HF}|\bar{H}|\mathrm{HF}\rangle & \langle \mathrm{HF}|\bar{H}|R_1\rangle \\ \langle L_1|\bar{H}|\mathrm{HF}\rangle & \langle L_1|\bar{H}|R_1\rangle \end{pmatrix} = \begin{pmatrix} E_0 & \boldsymbol{\eta}^{\mathrm{T}}\mathbf{r}_1 \\ \mathbf{l}_1^{\mathrm{T}}\boldsymbol{\Omega} & E_0 + W_{11} \end{pmatrix} \tag{1}$$

where

$$W_{11} = \langle \mathrm{L}_1|\bar{H}|\mathrm{R}_1\rangle - E_0 = \omega_1 + \sum_{\nu} \langle \mathrm{L}_1|\mathrm{R}_1|\nu\rangle \Omega_\nu \tag{2}$$

Full space Hamiltonian matrix

$$\mathbf{H}^{\mathrm{FS}} = \begin{pmatrix} \langle \mathrm{HF}|\bar{H}|\mathrm{HF}\rangle & \langle \mathrm{HF}|\bar{H}|R_1\rangle & \langle \mathrm{HF}|\bar{H}|\tilde{\nu}\rangle \\ \langle L_1|\bar{H}|\mathrm{HF}\rangle & \langle \mathrm{L}_1|\bar{H}|R_1\rangle & \langle L_1|\bar{H}|\tilde{\nu}\rangle \\ \langle \tilde{\mu}|\bar{H}|\mathrm{HF}\rangle & \langle \tilde{\mu}|\bar{H}|R_1\rangle & \langle \tilde{\mu}|\bar{H}|\tilde{\nu}\rangle \end{pmatrix} = \begin{pmatrix} E_0 & \boldsymbol{\eta}^T\mathbf{r}_1 & X_\nu \\ \mathbf{l}_1^T\boldsymbol{\Omega} & E_0 + W_{11} & Y_\nu \\ 0 & V_\mu & Z_{\mu\nu} + \delta_{\mu\nu}E_0 \end{pmatrix} \quad (3)$$

where

$$X_\nu = \langle \mathrm{HF}|\bar{H}|\tilde{\nu}\rangle = \eta_\nu - (\boldsymbol{\eta}^T\mathbf{r}_1)\mathrm{L}_{1\nu} \quad (4)$$

$$Y_\nu = \langle \mathrm{L}_1|\bar{H}|\tilde{\nu}\rangle = \langle \mathrm{L}_1|\bar{H}|\nu\rangle - (E_0 + W_{11})\mathrm{L}_{1\nu} \quad (5)$$

$$V_\mu = \langle \tilde{\mu}|\bar{H}|\mathrm{R}_1\rangle = \langle \mu|\bar{H}|\mathrm{R}_1\rangle - (E_0 + W_{11})\mathrm{R}_{1\mu} \quad (6)$$

$$Z_{\mu\nu} = \langle \tilde{\mu}|\bar{H}|\tilde{\nu}\rangle - \delta_{\mu\nu}E_0 = \langle \mu|\bar{H}|\nu\rangle - \delta_{\mu\nu}E_0 - \mathrm{R}_{1\mu}Y_\nu - (E_0 + W_{11})\mathrm{R}_{1\mu}\mathrm{L}_{1\nu} - V_\mu\mathrm{L}_{1\nu} \quad (7)$$

Multiple projected states

Reduced space Hamiltonian matrix

$$\mathbf{H}^{\mathrm{RS}} = \begin{pmatrix} \langle \mathrm{HF}|\bar{H}|\mathrm{HF}\rangle & \langle \mathrm{HF}|\bar{H}|\mathrm{R}_1\rangle & \dots & \langle \mathrm{HF}|\bar{H}|\mathrm{R}_N\rangle \\ \langle \mathrm{L}_1|\bar{H}|\mathrm{HF}\rangle & \langle \mathrm{L}_1|\bar{H}|\mathrm{R}_1\rangle & \dots & \langle \mathrm{L}_1|\bar{H}|\mathrm{R}_N\rangle \\ \vdots & \vdots & \ddots & \vdots \\ \langle \mathrm{L}_N|\bar{H}|\mathrm{HF}\rangle & \langle \mathrm{L}_N|\bar{H}|\mathrm{R}_1\rangle & \dots & \langle \mathrm{L}_N|\bar{H}|\mathrm{R}_N\rangle \end{pmatrix} = \begin{pmatrix} E_0 & \boldsymbol{\eta}^T\mathbf{r}_1 & \dots & \boldsymbol{\eta}^T\mathbf{r}_N \\ \mathbf{l}_1^{\mathrm{T}}\boldsymbol{\Omega} & E_0 + W_{11} & \dots & W_{1N} \\ \vdots & \vdots & \ddots & \vdots \\ \mathbf{l}_N^{\mathrm{T}}\boldsymbol{\Omega} & W_{N1} & \dots & E_0 + W_{NN} \end{pmatrix} \quad (8)$$

where

$$W_{IJ} = \langle \mathrm{L}_I|\bar{H}|\mathrm{R}_J\rangle - \delta_{IJ}E_0 = \delta_{IJ}\omega_I + \sum_\nu \langle \mathrm{L}_I|\mathrm{R}_J|\nu\rangle\Omega_\nu \quad (9)$$

Full space Hamiltonian matrix

$$\mathbf{H}^{\text{FS}} = \begin{pmatrix} \langle \text{HF}|\bar{H}|\text{HF}\rangle & \langle \text{HF}|\bar{H}|\text{R}_1\rangle & \dots & \langle \text{HF}|\bar{H}|\text{R}_N\rangle & \langle \text{HF}|\bar{H}|\tilde{\nu}\rangle \\ \langle \text{L}_1|\bar{H}|\text{HF}\rangle & \langle \text{L}_1|\bar{H}|\text{R}_1\rangle & \dots & \langle \text{L}_1|\bar{H}|\text{R}_N\rangle & \langle \text{L}_1|\bar{H}|\tilde{\nu}\rangle \\ \vdots & \vdots & \ddots & \vdots & \vdots \\ \langle \text{L}_N|\bar{H}|\text{HF}\rangle & \langle \text{L}_N|\bar{H}|\text{R}_1\rangle & \dots & \langle \text{L}_N|\bar{H}|\text{R}_N\rangle & \langle \text{L}_N|\bar{H}|\tilde{\nu}\rangle \\ \langle \tilde{\mu}|\bar{H}|\text{HF}\rangle & \langle \tilde{\mu}|\bar{H}|\text{R}_1\rangle & \dots & \langle \tilde{\mu}|\bar{H}|\text{R}_N\rangle & \langle \tilde{\mu}|\bar{H}|\tilde{\nu}\rangle \end{pmatrix} = \tag{10}$$

$$= \begin{pmatrix} E_0 & \boldsymbol{\eta}^T\mathbf{r}_1 & \dots & \boldsymbol{\eta}^T\mathbf{r}_N & X_\nu \\ \mathbf{l}_1^{\text{T}}\boldsymbol{\Omega} & E_0 + W_{11} & \dots & W_{1N} & Y_{1,\nu} \\ \vdots & \vdots & \ddots & \vdots & \vdots \\ \mathbf{l}_N^{\text{T}}\boldsymbol{\Omega} & W_{N1} & \dots & E_0 + W_{NN} & Y_{N,\nu} \\ 0 & V_{1,\mu} & \dots & V_{N,\mu} & Z_{\mu\nu} + \delta_{\mu\nu}E_0 \end{pmatrix} \tag{11}$$

where

$$X_\nu = \langle \text{HF}|\bar{H}|\tilde{\nu}\rangle = \eta_\nu - \sum_I (\boldsymbol{\eta}^T\mathbf{r}_I)\text{L}_{I\nu} \tag{12}$$

$$Y_{I,\nu} = \langle \text{L}_I|\bar{H}|\tilde{\nu}\rangle = \langle \text{L}_I|\bar{H}|\nu\rangle - \sum_J (E_0\delta_{IJ} + W_{IJ})\text{L}_{J\nu} \tag{13}$$

$$V_{I,\mu} = \langle \tilde{\mu}|\bar{H}|\text{R}_I\rangle = \langle \mu|\bar{H}|\text{R}_I\rangle - \sum_J \text{R}_{J\mu}(E_0\delta_{IJ} + W_{JI}) \tag{14}$$

$$Z_{\mu\nu} = \langle \tilde{\mu}|\bar{H}|\tilde{\nu}\rangle = \langle \mu|\bar{H}|\nu\rangle - \delta_{\mu\nu}E_0 - \sum_I \text{R}_{I\mu}Y_{I,\nu} - \sum_I V_{I,\mu}\text{L}_{I\nu} - \sum_{IJ} \text{R}_{I\mu}(E_0\delta_{IJ} + W_{IJ})\text{L}_{J\nu} \tag{15}$$

Algorithm and computational details

The full matrix eigenvalue equation in line 13 is solved using Davidson's algorithm. The initial vectors are set as:

$$\mathbf{x}_j = \begin{pmatrix} \mathbf{x}_j^{\text{RS}} \\ \mathbf{0} \end{pmatrix} \text{ for } j = 1, \dots, n_{\text{proj}} + 1 \text{ and } \mathbf{x}_j = \begin{pmatrix} \mathbf{0} \\ \mathbf{R}_{j-1} \end{pmatrix} \text{ for } j = n_{\text{proj}} + 2, \dots, n_{\text{excited}} + 1 \tag{16}$$

Algorithm 1 GCCSD algorithm

1: $k = 0$
2: $t^{[0]}$= guess
3: **while** (k < $\max_{\text{iteration}}$ and $\|\tilde{\Omega}\|_{L_2}$ > threshold) **do**
4: Solve the eigenvalue equation $\mathbf{A}\mathbf{r}_i^{[k]} = \omega_i^{[k]}\mathbf{r}_i^{[k]}, \quad i = 1, \ldots, n_{\text{excited}}$
5: Solve the eigenvalue equation $\mathbf{A}^{\text{T}}\mathbf{l}_i^{[k]} = \omega_i^{[k]}\mathbf{l}_i^{[k]}, \quad i = 1, .., n_{\text{excited}}$
6: Biorthonormalize $\mathbf{r}_i^{[k]}$ and $\mathbf{l}_i^{[k]}$
7: $t^{[k]} \leftarrow t^{[k]} - \hat{P}^{[k]}t^{[k]}$ ▷ $\hat{P}^{[k]} = \sum_{i=1}^{n_{\text{proj}}} \mathbf{r}_i^{[k]}\mathbf{l}_i^{[k]T}$
8: Construct $\Omega_\mu^{[k]}[t^{[k]}]$
9: Remove the projection $\tilde{\Omega}_\mu^{[k]} = \Omega_\mu^{[k]} - \hat{P}^{[k]}\Omega_\mu^{[k]}$
10: $t_\mu^{[k+1]} \leftarrow t_\mu^{[k]} - \tilde{\Omega}_\mu^{[k]}/\epsilon_\mu$ ▷ ϵ_μ is given by orbital energy differences
11: $k \leftarrow k + 1$
12: **end while**
13: Construct and diagonalize the reduced matrix $\mathbf{H}^{\text{RS}}$
14: Solve the eigenvalue equation $\mathbf{H}^{\text{FS}}\mathbf{x}_j = \mathcal{E}_j\mathbf{x}_j, \quad j = 1, \ldots, n_{\text{excited}} + 1$

Note that at the beginning is not necessary to solve the eigenvalue problems in lines 4-5 with very tight threshold. To significantly reduce the number of iterations needed and the overall computational costs, the threshold used for the residual at the k-th macroiteration is set to be $\min(10^{-2}, 5\|\tilde{\Omega}^{[k]}\|_{L_2})$ so that it improves following the convergence of the cluster amplitudes.

Another issue to consider regards the overall sign of the eigenvectors, which is random after the diagonalization in the subspace of guess vectors. This does not change the projector, as left and right vectors are later biorthogonalized, but it makes following the phase of the eigenvectors difficult to follow. To avoid this when restarting a calculation from a previous geometry, the sign of each eigenvector is assigned in such a way that the dot product between the current eigenvector and the respective eigenvector at the previous geometry is positive. As long as the geometries are sufficiently similar, this ensures that the overall sign of the vector is continuous for neighboring geometries but still allows any single component of the vector to change sign, going through zero.

Results on convergence and size extensivity

Non-interacting identical water molecules

Table S1: Ground state and excited state CCSD and GCCSD energies for a single water molecule and two identical non-interacting water molecules. All calculations are performed with aug-cc-pVDZ, with the second water molecule translated by 500 Bohr on both x and z. For a single water molecule, Δ E_1 refers to the first total symmetric state which is the third excited state and 3 states are included in the projector. For the case of two water molecules, Δ E_1 and Δ E_2 are the fifth and sixth excited states respectively and 6 states are included in the projector.

System	E_0/n_{H_2O}	Δ E_1	Δ E_2
CCSD 1 H_2O	-76.269497284	0.347280380	
GCCSD 1 H_2O	-76.269497286	0.347201630	
CCSD 2 H_2O	-76.269497284	0.347280380	0.347280381
GCCSD 2 H_2O	-76.269497286	0.347201701	0.347239236

Table S2: Water geometries in Bohr.

Atom	x	y	z
O	0.00000000	0.000000	-0.009000
H	0.00000000	1.515263	-1.058898
H	0.00000000	-1.515263	-1.058898
O	500.000000	0.000000	-500.009000
H	500.000000	1.515263	-501.058898
H	500.000000	-1.515263	-501.058898

Effect of the number of states in the projector

Table S3: Thymine energies in Hartree when changing the number of projected states. All calculations are performed with cc-pVDZ, converged up to $1 \cdot 10^{-10}$. FS refers to eigenvalues of the full space matrix and RS to the one of the reduced space, with a number to indicate the number of states included in the projector.

Method	E_0	E_1	E_2	E_3
CCSD	-452.9130722504	-452.7700019437	-452.7254480381	-452.7002632744
FS 1	-452.9130722505	-452.7700019491	-452.7254479439	-452.7002633747
RS 1	-452.9130722509	-452.7700019484		
FS 2	-452.9130738420	-452.7708068743	-452.7265615865	-452.7008285885
RS 2	-452.9130960357	-452.7708061397	-452.7265005712	
FS 3	-452.9130787502	-452.7711787685	-452.7268942411	-452.7006129360
RS 3	-452.9131081253	-452.7711813076	-452.7268576030	-452.7005857256
FS 4	-452.9130783759	-452.7711987402	-452.7269036334	-452.7006463304
RS 4	-452.9131083092	-452.7712007102	-452.7268674102	-452.7006197380
FS 5	-452.9130795921	-452.7711262607	-452.7269319103	-452.7005540834
RS 5	-452.9131103374	-452.7711291875	-452.7268949571	-452.7005300811

Table S4: Thymine GCCSD excitation energy differences in eV when changing the number of projected states. The ground state energies in the first column are expressed in Hartree and expressed with respect to the CCSD value as Δ GS = $E^{GS}_{method} - E^{GS}_{CCSD}$. All calculations are performed with cc-pVDZ, converged up to $1 \cdot 10^{-10}$.

System	Δ GS	Δ E_1	Δ E_2	Δ E_3
CCSD	-452.9130722504	3.893141343	5.105514866	5.790827195
GCCSD proj 1	-1.1e-10	3.893141198	5.105517434	5.790824466
GCCSD proj 2	-1.6e-6	3.871281376	5.075256981	5.775487524
GCCSD proj 3	-6.5e-6	3.861295178	5.066338547	5.781489285
GCCSD proj 4	-6.1e-6	3.860741537	5.066072787	5.780570396
GCCSD proj 5	-7.3e-6	3.862746898	5.065336425	5.783113657

Table S5: Thymine and a single He atom shifted by 500 Å on all directions, when projecting different numbers of states. All calculations are performed with cc-pVDZ, converged up to $1 \cdot 10^{-10}$. FS refers to the eigenvalues of the full space matrix and RS to the one of the reduced space, with a number to indicate the number of states included in the projector.

System	E_0-E^{He}_{CCSD}	E_1-E^{He}_{CCSD}	E_2-E^{He}_{CCSD}	E_3-E^{He}_{CCSD}
CCSD	-452.9130722504	-452.7700019437	-452.7254480381	-452.7002632744
FS 1	-452.9130722505	-452.7700019491	-452.7254479439	-452.7002633748
RS 1	-452.9130722509	-452.7700019484		
FS 3	-452.9130787502	-452.7711787685	-452.7268942411	-452.7006129360
RS 3	-452.9131081253	-452.7711813076	-452.7268576030	-452.7005857256
FS 5	-452.9130795921	-452.7711262607	-452.7269319103	-452.7005540834
RS 5	-452.9131103374	-452.7711291875	-452.7268949571	-452.7005300810

Table S6: Thymine geometry at the minimum in Angstrom.

Atom	x	y	z
C	1.626856184467	-0.090172437156	0.013282935761
C	-0.197850164697	1.572388519191	-0.033300408283
C	-0.740709833470	-0.739313238235	-0.079521724593
C	-1.176243220430	0.598835299691	-0.065959144242
C	-2.652953867469	0.918586250733	-0.094848197888
N	0.630784739942	-1.058855452370	-0.174427947644
N	1.164641231138	1.205510451408	-0.019672119665
O	2.794813959974	-0.394624876859	0.158035521208
O	-1.546761142159	-1.810516595404	-0.194583290615
H	1.881303447604	1.906943526328	0.115985843483
H	-0.412611083215	2.640305248447	-0.000321107921
H	0.924394117785	-1.993233441610	0.093943333488
H	-2.811463058403	2.006697839314	-0.031363123742
H	-3.180294992549	0.445499759315	0.751943675010
H	-3.121321727307	0.554762000579	-1.026259737912

Ethylene

Ethylene 2D scan

All 2D scans are run with the initial geometry, **g** and **h** vectors reported below. These vectors are originally determined in Hartree/Bohr and later used as displacement vectors in Bohr, defining a new geometry $\mathbf{r}_0 + \alpha\mathbf{g} + \beta\mathbf{h}$ from the initial geometry $\mathbf{r}_0$. For GCCSD the scheme used to cover the space was the one in Fig. S1a. For The CCSD results the scheme in Fig. S1b was chosen instead, to restrict the area where the flipped solution with negative excitation energy was obtained.

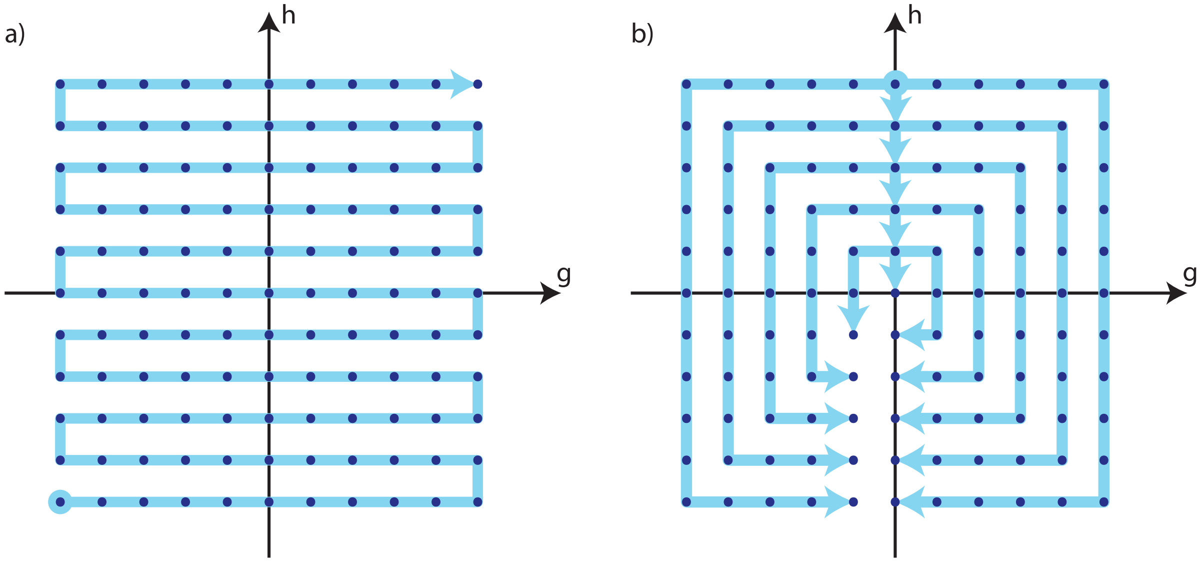

Figure S1: Graphical representation of the algorithms used to run the 2D scan.

Table S7: Ethylene ε-MECI geometry in Bohr.

Atom	x	y	z
H	1.999349425914	-0.910907722158	1.414804072229
C	1.165469892760	0.249256850813	-0.120813103612
H	2.556017310769	0.837765453705	-1.559399709943
C	-1.461989224355	0.680278349416	-0.470086114356
H	-2.617593462368	0.855685356721	1.279385377432
H	-1.641442915322	-1.523105689634	-0.544646412150

Table S8: Ethylene g vector in Hartree/Bohr.

Atom	x	y	z
H	0.007313466289	0.057147259497	0.037233885635
C	0.018065045039	0.010653044239	0.029770694182
H	-0.011045778402	-0.044246241553	-0.025640855466
C	-0.016011637809	-0.042775821534	0.029986440784
H	0.030212324793	-0.002500610868	0.030496622594
H	-0.028533419910	0.021722370219	-0.10184678772]

Table S9: Ethylene h vector in Hartree/Bohr.

Atom	x	y	z
H	0.009983729150	0.004279864574	0.006085526830
C	-0.059128405254	-0.031406366244	0.011698042309
H	-0.000072261588	-0.004346978757	-0.005302673058
C	0.013554961398	0.069990614294	-0.037047741578
H	0.008830292504	-0.027241876753	0.014224759752
H	0.027122442072	-0.011417198126	0.010473940680

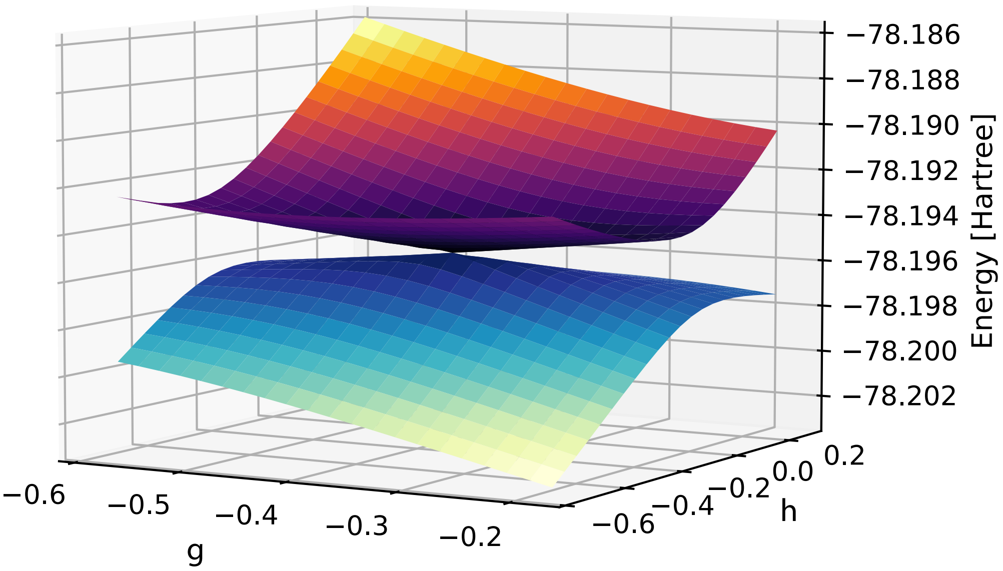

Figure S2: The GCCSD potential energy surfaces of S_0 and S_1 in ethylene. The basis is aug-cc-pVDZ and the energies are the total enegies expressed in Hartree.

Ethylene circle

The **g** and **h** vectors are the same ones as in the previous section. The center of the circle is defined as $(\mathrm{g}, \mathrm{h}) = (-0.385, -0.13)$ from the ε-MECI, which is very close to the intersection for GCCSD, and the radius is radius $r = \sqrt{g^2 + h^2} = 0.8$. For the GCCSD calculations in

Fig. 4, the first point of the circle is $(\mathrm{g}, \mathrm{h}) = (0.8, 0.0)$, set as 0°, and two full rotations are completed restarting each calculation from the previous geometry. For the CCSD results in Fig. 5a, the first point of the circle is $(\mathrm{g}, \mathrm{h}) = (0.0, 0.8)$, corresponding to 0°, and the scan was run restarting for half a circle in one direction and then restarted from the initial point for half a circle in the opposite direction. The second CCSD circle in Fig. 5c was run following the same procedure but with the initial point in $(\mathrm{g}, \mathrm{h}) = (0.0, -0.8)$.

Ethylene extensivity and convergence

The structures used in Tables 1-2 as ethylene A, B, and C are obtained from the CCSD circle defined in the previous section, corresponding to the points at 0°, 3° and 6°. The corresponding geometries are reported below, with B and C translated by 1000 Å on y and z respectively.

Conversion factor: 1Å = 1.8897259886 Bohr

Table S10: Ethylene A geometry in Angstrom.

Atom	x	y	z
H	1.0600599515526221	-0.4921570182186516	0.7432539625898773
C	0.5920957988879612	0.11859558726990083	-0.06584929946247757
H	1.3548109808694992	0.4507996323673442	-0.8218550101479879
C	-0.7655834382281663	0.39351776753311646	-0.2680033422675551
H	-1.3881953930153716	0.44366010709388964	0.6758518295627777
H	-0.8531848120811245	-0.8144664010539469	-0.2637513912751824

Table S11: Ethylene B geometry in Angstrom.

Atom	x	y	z
H	1.0598921220967552	999.5065743441498	0.7424254780268561
C	0.5917298546240902	1000.11837777963987291	-0.0665156858139174
H	1.355055753036723	1000.4517824740106923	-0.821283834929158
C	-0.7652365486675872	1000.39442490172403727	-0.2686462276042272
H	-1.3888699003363099	1000.4437313156848888	0.6751678936041264
H	-0.8525683614590025	999.1850589421331	-0.26150095078339364

Table S12: Ethylene C geometry in Angstrom.

Atom	x	y	z
H	1.0597131680251948	-0.4946957823769698	-999.2584077970613
C	0.5914335230593863	0.11819701139648099	-1000.06719381947529221
H	1.3552999381492163	0.45276766577214966	-1000.8207080723028558
C	-0.764906338412678	0.3952483358827204	-1000.2692443624734343
H	-1.3895528051134067	0.44383393922191544	-999.32553067344
H	-0.8519850720229274	-0.8154011657277284	-1000.2592688320343005

Table S13: **A detailed breakdown of the individual contributions to the total time for the case of an ethylene molecule, as described in Table 2. The timings are wall times in seconds on an Intel Xeon Gold 6342 using 24 cores. For GCCSD, the projection of the omega, the projection of the amplitudes and the construction and diagonalization of the reduced matrix are all included in the Ground state section.**

CCSD	Section	GCCSD
1.76s	Reference wavefunction	1.56s
10.94s	Cholesky decomposition	6.51s
9.61s	Ground state	9.72s
16.78s	Right eigenvectors	32.88s
17.63s	Left eigenvectors	33.24s
-	Full matrix eigenvectors	12.22s
56.96s	Total time	96.33s

For CCSD, the number of iterations to determine the right eigenvectors of the Jacobian was 33, for a total of 87 Jacobian transformations. The number of iterations to determine the left eigenvectors of the Jacobian was 33, for a total of 84 Jacobian transpose transformations.
For GCCSD, the number of iterations to determine the right eigenvectors of the Jacobian was 101, for a total of 244 Jacobian transformations. The number of iterations to determine the left eigenvectors of the Jacobian was 104, for a total of 231 Jacobian transpose transformations. The number of iterations to determine the right eigenvectors of the full matrix was 27, for a total of 69 full matrix transformations.
We point out that for GCCSD all 3 right and left eigenvectors are determined and updated when solving for the ground state, even though only one state is projected. This was done to show a fair comparison between the methods, as all 3 states are required for CCSD. In GCCSD, only the eigenvectors that need to be projected are required during the ground state procedure, resulting in a decrease in computational cost. All the remaining eigenvalues and eigenvectors are later obtained through the final solution of the full matrix eigenvalue problem. We also note that this final step of the algorithm presents a computational cost and scaling with system size that is nearly identical to solving the right eigenvalue problem for the Jacobian in standard CCSD, since the complexity of the contributions is identical

and the size of the problem is increased only by the number of projected states.

Thymine 2D scan

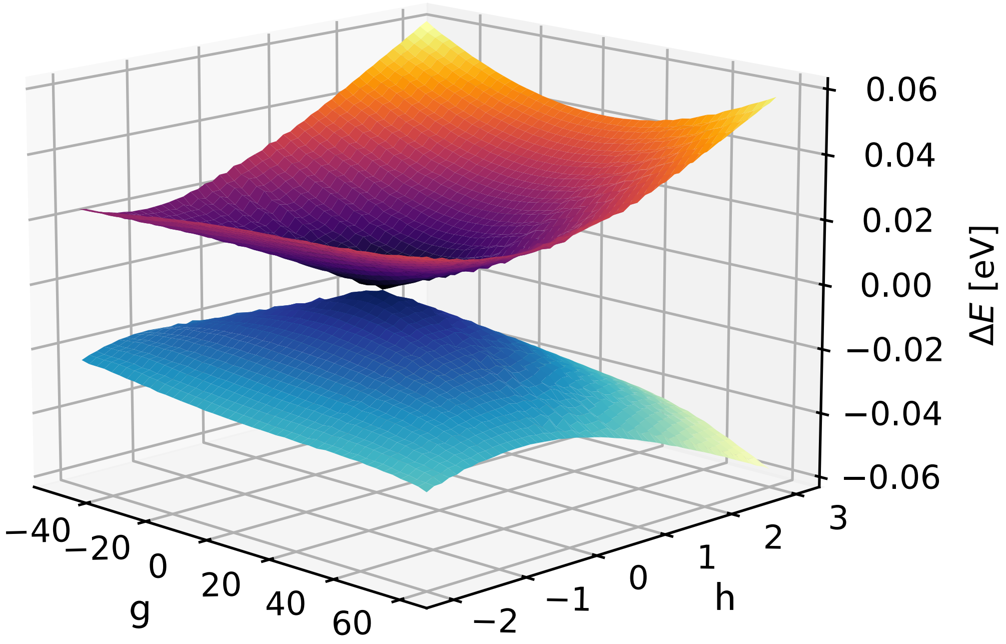

Figure S3: SCCSD potential energy surfaces of S_1 and S_2 in thymine with cc-pVDZ basis. All energies are plotted in eV, relative to the average $\frac{1}{2}(E_1 + E_2)$ for each point. The plot shows the same region of Fig. 3c in Ref. 4, which is larger than what shown in Fig. 6. The axes have also been rescaled to be consistent with Fig. 6. Note that the thresholds for the calculations used in the reference are different, which results in visible noise.

Table S14: Thymine initial geometry in Bohr.

Atom	x	y	z
C	3.024632508678	-0.197939565118	-0.043506106319
C	-0.379163249776	2.993201825994	-0.376843901976
C	-1.584772946934	-1.537050600849	-0.174652122376
C	-2.22289716323	1.092687974969	-0.019993160668
C	-4.942441756804	1.880988047973	0.295884973444
N	1.383965794231	-2.10204230652	-0.134678067251
N	2.112941375643	2.387375342191	0.152606749372
O	5.327286048654	-0.549667905189	0.112112165406
O	-2.956702928079	-3.541736963203	0.113404299659
H	3.56372918388	3.213977204429	0.959418384849
H	-1.197624746346	4.870276855875	-1.007782429014
H	2.125362704196	-3.672844271999	-0.139754412083
H	-5.173960594864	3.777158444758	1.238505943709
H	-6.247239158792	0.021538888608	0.656427604651
H	-5.484297294198	2.089178080965	-1.738950890195

Table S15: Thymine g vector in Hartree/Bohr.

Atom	x	y	z
C	-0.0005798127253398536	-0.0006105588384348458	-0.0003687934628399069
C	0.0003818164857299036	-0.0002872196909549275	-0.00020855092277994734
C	-0.000807956004604796	0.00047609943831487984	-0.0002163023882049454
C	-0.00026859391788993216	0.00032664710958991755	-8.684068250997807e-05
C	0.0004941927063798752	0.0008342066308997893	0.0006916614546198255
N	0.0024098330181643915	-0.002301077258679419	0.00022497031079994322
N	0.00026493162539493315	0.0012347579959946884	0.0005719552842648555
O	0.00026468183472493317	-0.00011783293740497025	5.885903984998514e-05
O	-0.00025493002853493565	-0.00037689204538490486	2.6315375574993355e-05
H	-0.0003681107651799071	-0.0008153142290797942	-0.00019227043060995145
H	-0.00051518556615487	0.00024671446873993774	-0.00016483332544495842
H	-0.0007069723990748215	0.002157801584334554	4.683741952998818e-05
H	0.0001180527907449702	0.00046875080507488163	-5.661090652498571e-05
H	-0.0005499230343048612	-0.0009651818081297563	-0.00026794109715993234
H	0.00011797597996497022	-0.0002709012248249316	-5.8455668569985244e-05

Table S16: Thymine h vector in Hartree/Bohr.

Atom	x	y	z
C	0.00021457107071499082	0.001803226261795447	9.749523596998285e-05
C	-0.0010134839562702467	-0.0009720296212803169	0.0004611740828001079
C	0.0016994394176503926	0.0028170744749808733	-0.00037253367877013453
C	0.00038991867326007985	0.0004750303736451777	-6.875357674503058e-05
C	-0.0002448460136600101	4.055406735511435e-05	-0.0004715577597300515
N	-0.00039165567384481694	-0.0009536981321605591	5.521516118504366e-05
N	0.001120233510195357	-0.0005743336152900145	-0.000284505319725012
O	-0.0002510358241650401	-0.0005482676678901734	-2.513796076000004e-05
O	-0.0009858319883903168	-0.001872929150235589	0.00039741206067511834
H	-0.00022507579131511045	-0.0001022527671401299	-7.556058939004552e-05
H	-0.00010585761811509402	-6.751805858498892e-05	0.00013027901358001745
H	-1.3309690020090808e-05	-7.338529004736723e-06	8.97661201500836e-06
H	1.620964079001921e-05	-8.850160360996796e-05	3.125465966500208e-05
H	-8.820500490509318e-05	5.681394686489774e-05	1.355151105497097e-05
H	-0.00012105746345002055	-5.786971995034995e-06	0.00010269838165002255

2,4-Cyclohexadien-1-ylamine 2D scan

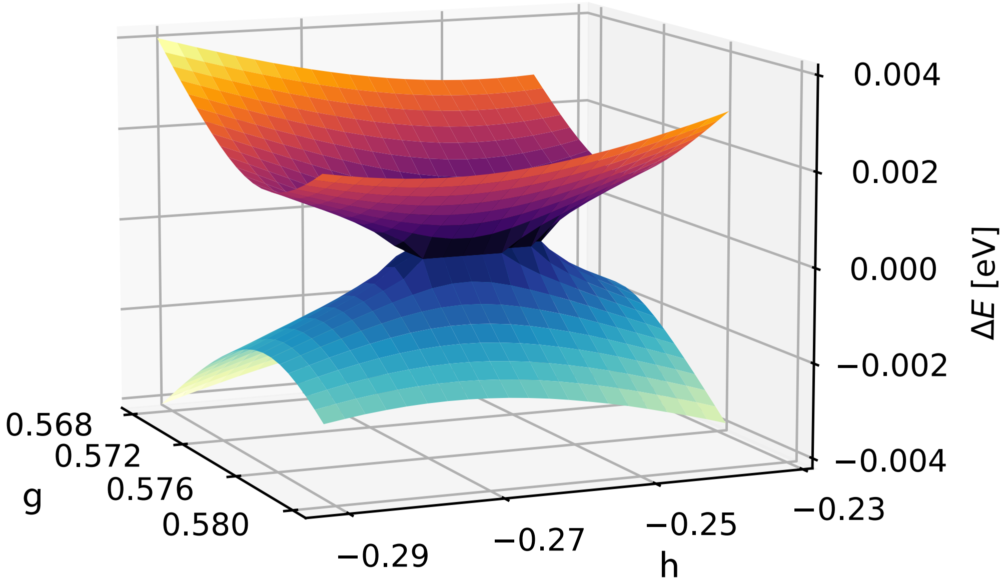

Figure S4: The GCCSD potential energy surfaces of S_0 and S_1 in 2,4-cyclohexadien-1-ylamine with cc-pVDZ in the region very close to the intersection. The energies for each point are plotted in eV relative to the average energy $\frac{1}{2}(E_0 + E_1)$.

Table S17: 2,4-Cyclohexadien-1-ylamine initial geometry in Bohr.

Atom	x	y	z
N	2.485229105603	0.547832318017	-1.016626362773
C	-1.020180309090	-2.514973398583	-1.130137584081
C	0.000000000000	0.000000000000	0.000000000000
C	-0.152827830887	-4.842609039125	0.243271218683
C	0.000000000000	0.000000000000	2.948948714717
C	0.393673950618	-4.812906418385	2.772429176890
C	0.638744772120	-2.397590397164	4.027981665337
H	3.068814728580	2.219087393160	-0.340105507921
H	3.743103862610	-0.735412398662	-0.407797247778
H	-0.597826785001	-2.656733416686	-3.131827502962
H	-1.241628965952	1.485927944692	-0.652347722798
H	-3.073407175968	-2.431583674518	-0.951269929831
H	-0.051896980885	-6.598968101875	-0.785039323552
H	-2.020803718174	-1.042080601584	3.045064771778
H	0.977265443085	-6.512166056261	3.733568986966
H	1.430957011893	-2.465032088650	5.918242701070

Table S18: 2,4-Cyclohexadien-1-ylamine g vector in Hartree/Bohr.

Atom	x	y	z
N	0.004971062452	-0.000006312063	-0.008307843382
C	-0.016216875814	-0.011028497968	0.013811359333
C	0.028843656521	0.052451171540	0.008092929065
C	-0.002450100320	-0.007281793260	-0.010822870907
C	-0.081339063409	-0.074869115270	-0.049735972788
C	-0.013448003251	0.032461991297	0.014876665117
C	0.068149686093	-0.043067069541	0.067549127712
H	0.000405335195	-0.001041323709	-0.002325561626
H	-0.001324788614	-0.000710206799	-0.000031584236
H	0.003448251125	0.006602895615	0.002116067133
H	-0.001001097065	-0.000044020470	-0.002328844295
H	0.003064563939	0.003521849557	-0.003645421539
H	0.001814047671	0.001543968627	-0.001141347406
H	-0.009685201838	0.038125491323	-0.021492693576
H	-0.000178824202	0.003941613764	0.002797133980
H	0.014947351518	-0.000600642643	-0.009411142586

Table S19: 2,4-Cyclohexadien-1-ylamine h vector in Hartree/Bohr.

Atom	x	y	z
N	0.002487591355	0.001059423611	-0.001229774795
C	0.001417281298	0.007961593644	0.001946831613
C	0.002788554845	-0.012082019030	-0.001934955082
C	0.000037767487	-0.003593455041	0.002688939505
C	-0.010315266883	-0.006727478406	-0.008749189841
C	0.018041726684	0.000359400262	-0.009740084068
C	0.007161006545	-0.003086550689	-0.006418446171
H	0.000523768377	0.000163090505	0.000341198029
H	0.000201834734	0.000043417177	-0.000047450886
H	0.000287627165	0.001485980656	0.000635812663
H	0.000112190184	0.000739350784	0.000996160267
H	-0.001339158720	-0.002603931746	-0.001285345959
H	-0.003490679658	-0.000662035975	0.000729035443
H	-0.004741496715	0.016752015594	0.017562156627
H	-0.001039863972	-0.000167499259	0.000074916208
H	-0.012149841791	0.000529111955	0.004488870984

References

(1) Sun, Q.; Berkelbach, T. C.; Blunt, N. S.; Booth, G. H.; Guo, S.; Li, Z.; Liu, J.; McClain, J. D.; Sayfutyarova, E. R.; Sharma, S. et al. PySCF: the Python-based simulations of chemistry framework. *Wiley Interdisciplinary Reviews: Computational Molecular Science* **2018**, *8*, e1340.

(2) *https://github.com/MarcusTL12/SpinAdaptedSecondQuantization.jl*

(3) Folkestad, S. D.; Kjønstad, E. F.; Myhre, R. H.; Andersen, J. H.; Balbi, A.; Coriani, S.; Giovannini, T.; Goletto, L.; Haugland, T. S.; Hutcheson, A. et al. eT 1.0: An open source electronic structure program with emphasis on coupled cluster and multilevel methods. *J. Chem. Phys.* **2020**, *152*, 184103.

(4) Kjønstad, E. F.; Fajen, O. J.; Paul, A. C.; Angelico, S.; Mayer, D.; Gühr, M.; Wolf, T. J.; Martínez, T. J.; Koch, H. Photoinduced hydrogen dissociation in thymine predicted by coupled cluster theory. *Nat. Commun.* **2024**, *15*, 10128.
